# Comparison of Drug-Induced Sleep Endoscopy and Natural Sleep Endoscopy in the Assessment of Upper Airway Pathophysiology During Sleep: Protocol and Study Design

**DOI:** 10.3389/fneur.2021.768973

**Published:** 2021-12-07

**Authors:** Karlien Van den Bossche, Eli Van de Perck, Andrew Wellman, Elahe Kazemeini, Marc Willemen, Johan Verbraecken, Olivier M. Vanderveken, Daniel Vena, Sara Op de Beeck

**Affiliations:** ^1^Department of ENT and Head and Neck Surgery, Antwerp University Hospital, Edegem, Belgium; ^2^Faculty of Medicine and Health Sciences, University of Antwerp, Wilrijk, Belgium; ^3^Division of Sleep and Circadian Disorders, Brigham and Women's Hospital and Harvard Medical School, Boston, MA, United States; ^4^Multidisciplinary Sleep Disorders Center, Antwerp University Hospital, Edegem, Belgium

**Keywords:** diagnosis, endotyping, OSA (obstructive sleep apnea), personalized medicine, upper airway obstruction

## Abstract

**Study Objectives:** Obstructive sleep apnea (OSA) is increasingly recognized as a complex and heterogenous disorder. As a result, a “one-size-fits-all” management approach should be avoided. Therefore, evaluation of pathophysiological endotyping in OSA patients is emphasized, with upper airway collapse during sleep as one of the main features. To assess the site(s) and pattern(s) of upper airway collapse, natural sleep endoscopy (NSE) is defined as the gold standard. As NSE is labor-intensive and time-consuming, it is not feasible in routine practice. Instead, drug-induced sleep endoscopy (DISE) is the most frequently used technique and can be considered as the clinical standard. Flow shape and snoring analysis are non-invasive measurement techniques, yet are still evolving. Although DISE is used as the clinical alternative to assess upper airway collapse, associations between DISE and NSE observations, and associated flow and snoring signals, have not been quantified satisfactorily. In the current project we aim to compare upper airway collapse identified in patients with OSA using endoscopic techniques as well as flow shape analysis and analysis of tracheal snoring sounds between natural and drug-induced sleep.

**Methods:** This study is a blinded prospective comparative multicenter cohort study. The study population will consist of adult patients with a recent diagnosis of OSA. Eligible patients will undergo a polysomnography (PSG) with NSE overnight and a DISE within 3 months. During DISE the upper airway is assessed under sedation by an experienced ear, nose, throat (ENT) surgeon using a flexible fiberoptic endoscope in the operating theater. In contrast to DISE, NSE is performed during natural sleep using a pediatric bronchoscope. During research DISE and NSE, the standard set-up is expanded with additional PSG measurements, including gold standard flow and analysis of tracheal snoring sounds.

**Conclusions:** This project will be one of the first studies to formally compare collapse patterns during natural and drug-induced sleep. Moreover, this will be, to the authors' best knowledge, the first comparative research in airflow shape and tracheal snoring sounds analysis between DISE and NSE. These novel and non-invasive diagnostic methods studying upper airway mechanics during sleep will be simultaneously validated against DISE and NSE.

**Clinical Trial Registration:** www.ClinicalTrials.gov, identifier: NCT04729478.

## Introduction

Obstructive sleep apnea (OSA) is a highly prevalent disorder affecting almost one billion individuals worldwide (1). This entity can be best described as a heterogenous condition with recurrent upper airway collapse provoking nocturnal breathing cessation (2). The pathophysiology of OSA is multifactorial and varies considerably between individuals (3). One of the key determinants is an anatomically narrow and collapsible upper airway (4, 5). However, non-anatomical traits are also important contributors to OSA pathogenesis, including an ineffective pharyngeal muscle response during sleep, an oversensitive ventilatory control system (i.e., high loop gain), and a low arousal threshold (6). The PALM concept combines these pathophysiological traits on a three-point scale (passive critical closing pressure, arousal threshold, loop gain, and muscle responsiveness), enabling categorization of patients into three categories: PALM scale 1 consists of patients with a high collapsible upper airway, necessitating a major anatomic intervention. PALM scale 2 includes patients with a moderate collapsibility who are candidates for an anatomic or non-anatomic intervention or a combination of both according to the other OSA traits, and PALM scale 3 is constituted of patients with predominantly non-anatomic traits and overall a less severe OSA (6). This research project will focus on the anatomical OSA traits, with a particular focus on the site of upper airway collapse.

The standard treatment for OSA is to pneumatically splint the upper airway throughout the respiratory cycle using continuous positive airway pressure (CPAP) (7). However, CPAP-therapy is often poorly tolerated with non-adherence rates ranging between 46 and 83% (8). Other treatment modalities, such as mandibular advancement devices (9), pharyngeal surgery (10), and upper airway stimulation (11) produce beneficial results in many cases but are limited by a variable efficacy. Thus, in light of the complex and heterogenous pathophysiology of OSA, treatment should be individually tailored according to various phenotypic traits (12–14).

At present, endoscopy-based methods are the clinical mainstay of outcome prediction and patient selection, allowing to identify the sites and patterns of upper airway collapse (15). The gold standard method, natural sleep endoscopy (NSE), visualizes upper airway dynamics using a pediatric bronchoscope during normal sleep (16). However, due to the labor-intensive and time-consuming nature of NSE, including overnight measurements, drug-induced sleep endoscopy (DISE) was developed as an alternative method for routine clinical practice (17). Using sedation to induce a sleep-like state, DISE offers the advantage of being performed during daytime hours in a high-volume setting (18). In the last two decades, DISE has gained great popularity for assessing the sites and patterns of upper airway collapse in patients with OSA.

Collapse can occur at multiple levels along the pharynx, including the soft palate, oropharynx, tongue base, and epiglottis. The absence of complete concentric collapse at the level of the palate (CCCp) and the presence of tongue base collapse during DISE may be positive predictors for therapeutic success with hypoglossal nerve stimulation therapy and mandibular advancement devices (19–21). The same favorable association between tongue base collapse and mandibular advancement device outcome was found by a recent study using NSE (22). Conversely, epiglottis collapse during DISE may negatively affect the outcome of mandibular advancement devices, CPAP, and upper airway surgery (23). However, this collapse type may respond well to changes in body position from supine to lateral as seen during both DISE and NSE (23, 24).

A recent literature review by our research group concluded that velar collapse is seen during NSE in 58.8% of patients, tongue base collapse in 43.2%, lateral wall collapse in 29.9%, and epiglottis collapse in 22.4% (25). Drug-induced sleep endoscopy studies in the past reported comparable findings with the highest prevalence of collapse at the level of the soft palate (81.0%), followed by the tongue base (46.6%), hypopharynx (38.7%), and oropharynx (21.9%) (15).

Park et al. recently explored the differences in obstruction patterns between DISE using midazolam and NSE (26). They demonstrated significant concordance in epiglottis collapse (92.3%), lateral wall collapse (88.5%), and velar collapse (76.9%). However, the agreement in tongue base collapse was relatively low (69.2%). Compared to NSE, CCCp was observed slightly more often during DISE. Regarding the extent of obstruction, higher obstruction grades were present during DISE observations, with more sites in need of treatment, in comparison to NSE. Overall, these findings would suggest that DISE is a reliable test. However, this study only included a small patient sample in the supine position.

An evolving method to non-invasively determine the sites of upper airway collapse is airflow shape analysis. Specific airflow features have been previously validated by using DISE and NSE observations (27–31). Negative effort dependence (NED) as a flow limitation characteristic is quantified as the percentage reduction in inspiratory flow from peak to plateau associated with increasing respiratory effort ([*flow*_*peak*_ − *flow*_*plateau*_]/*flow*_*peak*_). In a preliminary assessment of correlations between flow measurements and upper airway collapse sites during DISE, the authors observed that the presence of epiglottis collapse was associated with a higher NED compared to the absence of epiglottis collapse or collapse at other pharyngeal sites (30). This is in line with previous findings of Genta et al., who also found that tongue base collapse is associated with a low NED, and isolated palatal and lateral wall collapse with a moderate NED (27). Using a machine learning algorithm, Azarbarzin et al. expanded this work by developing a model that discriminated epiglottis from non-epiglottis collapse with 84% cross-validated accuracy (29). The main distinctive parameters of this model were the discontinuity index (slope of the steepest line fitted to the inspiratory airflow signal) and inspiratory jaggedness (extent of deviation from a flat line during inspiration). The same research group also identified expiratory flow limitation (recognizable as pinching of the airflow signal) as the hallmark of palatal prolapse (29). According to recent evidence, this pinching feature is associated with a negative response to mandibular advancement device treatment, indicating the potential of this non-invasive assessment (32).

Different parts of the upper airway may generate different snoring sounds. Therefore, acoustic analysis of tracheal snoring sounds could be a useful indicator of the site of obstruction in patients with OSA by determining the number of snore events, the intensity of snoring, the sound frequencies, and/or the spectrogram (33, 34). Liistro et al. were the first in 1991 who examined the relationship between snoring characteristics and the anatomical site of collapse by using a supraglottic pressure catheter (35). A study of Xu et al. showed a difference in sound spectrum between upper and lower-level obstructive apneas using esophageal and oropharyngeal pressure measurements. An obstruction level above the free margin of the soft palate produced a characteristic frequency in the low frequency domain and an obstruction level below the free margin of the soft palate in the high frequency domain (36). However, most studies have not used direct visualization of the upper airway with DISE. Recent studies using DISE have shown similar associations between the sites of collapse and certain frequency bands (37, 38). Palatal snoring was identified at low frequencies (137, 105–189 Hz), tongue base snoring at high frequencies (1,243, 1,215–1,277 Hz), and epiglottic snoring in a mid-frequency range (490, 331–510 Hz) (38). Multilevel obstruction during DISE might be associated with complex snoring sounds, expressing as an irregular pattern of snoring (37) or a high maximal intensity of low-frequency snoring sounds (≥60 dB) (33). Koo et al. stated that the spectrograms and frequencies of snoring sounds of drug-induced sleep did not differ significantly from those of natural sleep (39). Nevertheless, induced sleep might show a slightly higher intensity than natural sleep, predominantly on a retro-lingual obstruction level, and might be more irregular in comparison with natural sleep on spectrogram (34, 38–41). Sebastian et al. used an automated technique from audio signal recordings to identify the primary site of upper airway collapse. The automated system showed a high accuracy for identifying tongue and non-tongue related collapse (macro-average recall of 80%) but with only low unweighted average recalls for classifying all sites (34). A review of Penzel and Sabil supports the hypothesis of tracheal sound analysis to be a surrogate for respiratory flow (42). In a more recent study of the same research group with a small sample size (*n* = 32), the detection of apneas was compared using four different methods of airflow signals, concluding tracheal sounds to be a possible future substitute for oral thermistors (43). However, more studies utilizing endoscopic visualization of the upper airway during sleep, are necessary to validate this hypothesis. To conclude, tracheal snoring sounds analysis may be used as a useful screening tool to determine the site of obstruction during sleep.

In the current project, we aim to compare endoscopic findings of upper airway collapse as well as flow and tracheal snoring sounds analysis between natural and drug-induced sleep in patients with OSA.

## Materials and Methods

### Study Objectives

The study aims are as follows:

1. To compare upper airway collapse observed during NSE and DISE in patients with OSA.
2. To compare the airflow shapes caused by the different sites of collapse, between NSE and DISE.
3. To compare the acoustics of snores (i.e., tracheal snoring sounds) generated by the different sites of upper airway collapse between NSE and DISE.
4. To validate previously developed models for predicting the sites of collapse from airflow and snoring sounds and to compare prediction performance between NSE and DISE (27–31, 44, 45).

### Patient Recruitment

Regarding all four aims, patients participating in this study must follow the inclusion and exclusion criteria as listed in Table 1.

**Table 1:** Eligibility criteria.

Inclusion criteria	Age: 18 years or older BMI ≤35 kg/m^2^ Diagnosis of OSA with a baseline AHI ≥15/h based on full PSG Capability of giving informed consent and willingness to undergo NSE and DISE
Exclusion criteria	Central sleep apnea (defined as central AHI ≥30% of total AHI) Inability to sleep in a supine position due to a medical condition Inabilty of the patient to understand and/or comply to the study procedures Neuromuscular disorders or craniofacial anomalies affecting the upper airway Sedative medication use (opioids and muscle relaxants) Active psychiatric disorders (psychotic illness, major depression, anxiety attacks, excessive alcohol, or drug use) Severe or decompensated cardiac or respiratory diseases Contra-indications for DISE: fitness for general anesthesia (ASA >3), allergy to sedative agent(s), and an expected extremely difficult airway Pregnancy or willing to become pregnant

*AHI, apnea-hypopnea index; BMI, body mass index; DISE, drug-induced sleep endoscopy; NSE, natural sleep endoscopy; OSA, obstructive sleep apnea; PSG, polysomnography*.

To achieve the objectives, subjects will be prospectively recruited at the Department of Otorhinolaryngology in the Antwerp University Hospital and at the Department of Sleep and Circadian Disorders in the Brigham and Women's Hospital between March 1, 2021 and September 30, 2022. Every year, approximately 400 patients undergo a DISE at both departments combined. As a result, we expect to be able to include 40 participants during this time frame. Being a pilot study, no formal sample size calculations can be performed. Power analyses will be performed retrospectively to include additional patients if necessary.

### Study Design

**Figure 1:**
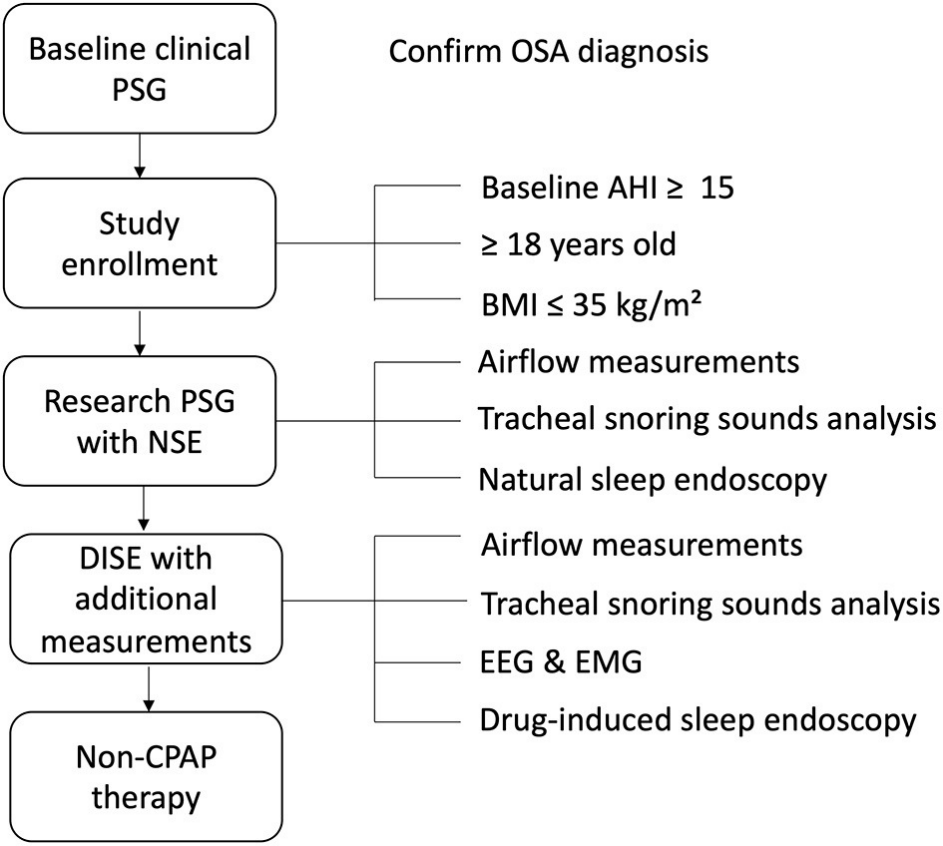
Generic study protocol. AHI, apnea-hypopnea index; BMI, body mass index; CPAP, continuous positive airway pressure; DISE, drug-induced sleep endoscopy; EEG, electroencephalography; EMG, electromyography; NSE, natural sleep endoscopy; OSA, obstructive sleep apnea; PSG, polysomnography.

**Figure 2:**
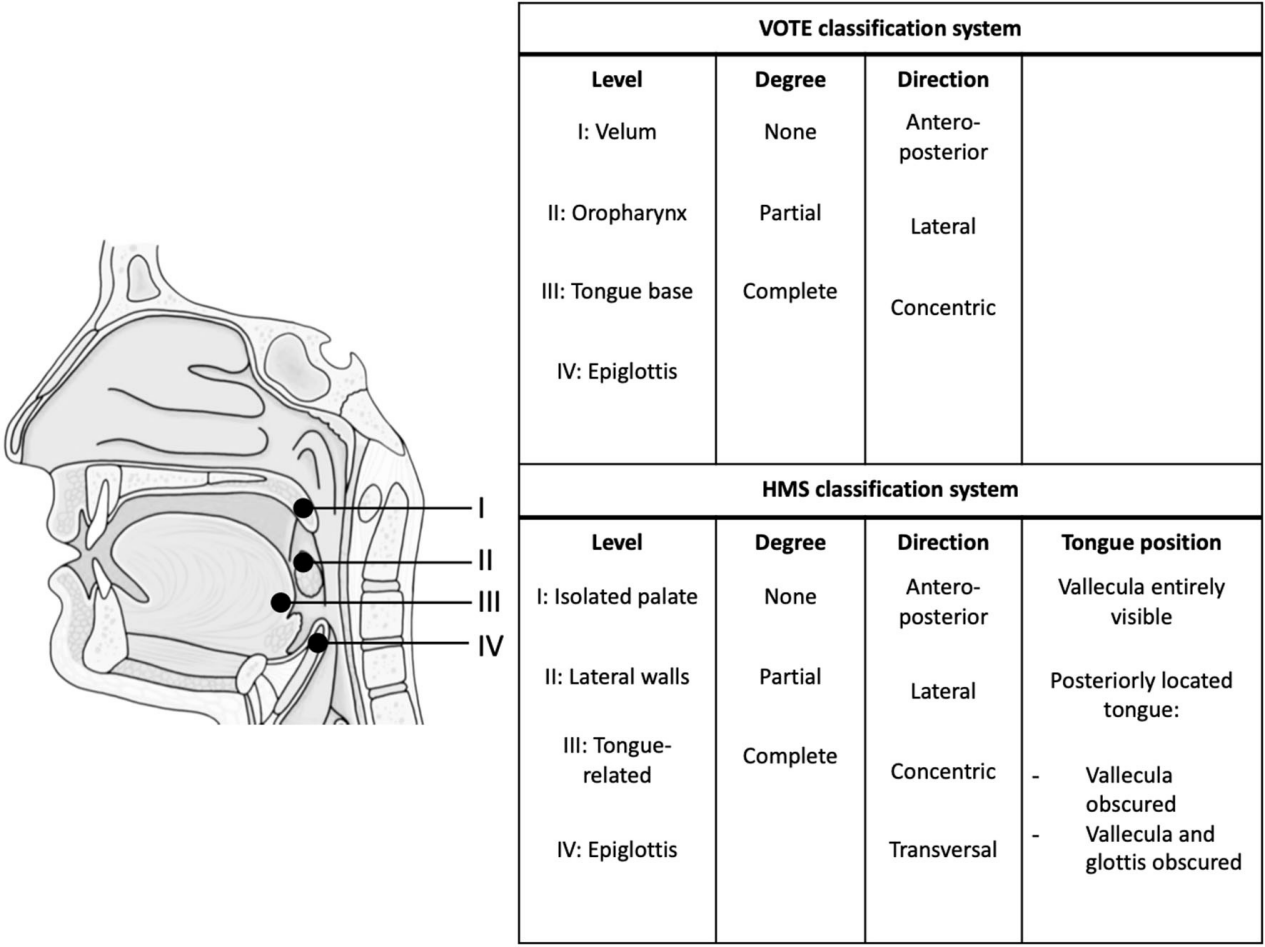
Endoscopic classification systems. HMS, Harvard Medical School. VOTE classification system (26, 46). HMS classification system (22, 27, 29).

This study is a blinded prospective comparative multicenter cohort study. The study population will consist of adult patients with a recent diagnosis of OSA [baseline apnea-hypopnea index (AHI) ≥15/h based on polysomnography (PSG)]. After receiving informed consent, eligible patients will undergo a research PSG (with NSE) and a subsequent DISE procedure within 3 months (Figure 1). The research PSG will be identical to the clinical PSG (i.e., overnight sleep in a sleep laboratory with standard measurements), except that it includes NSE, as well as airflow measurements using a pneumotachometer, and acoustic measurements of snoring using a microphone placed over the trachea. For the DISE procedure, the patient is lying supine in a semi-dark and silent operating theater. An intravenous bolus injection of midazolam 1.5 mg will be used to induce sleep. Maintenance of sedated sleep will be obtained by a target-controlled infusion of propofol (2.0–3.0 μg/ml). Scoring of obstruction sites (for DISE and NSE) will be done using two standardized scoring systems as previously described by our research team (Figure 2) (25). Different ear, nose, throat (ENT) surgeons within the research team will perform and score the DISE and NSE of the same participating patient to ensure that all investigations are scored in a blinded fashion. During DISE, we will also use electroencephalography (EEG) and electromyography (EMG) measurements to enable scoring of sleep stages, and to control for them in our comparison of DISE and NSE.

### Study Materials

A type I full-night PSG will be performed using standard sleep study equipment, following latest American Academy of Sleep Medicine (AASM) guidelines (47, 48). Classic PSG consists of EEG channels (prefrontal, central, and occipital leads with a reference lead to the mastoids), three surface EMG channels measuring the activity of the submentalis and the bilateral tibialis anterior muscles, two electro-oculography (EOG) channels, one electrocardiography (ECG) channel, oxygen saturation measurement, a position detector and measurement of thoraco-abdominal movements.

**Figure 3:**
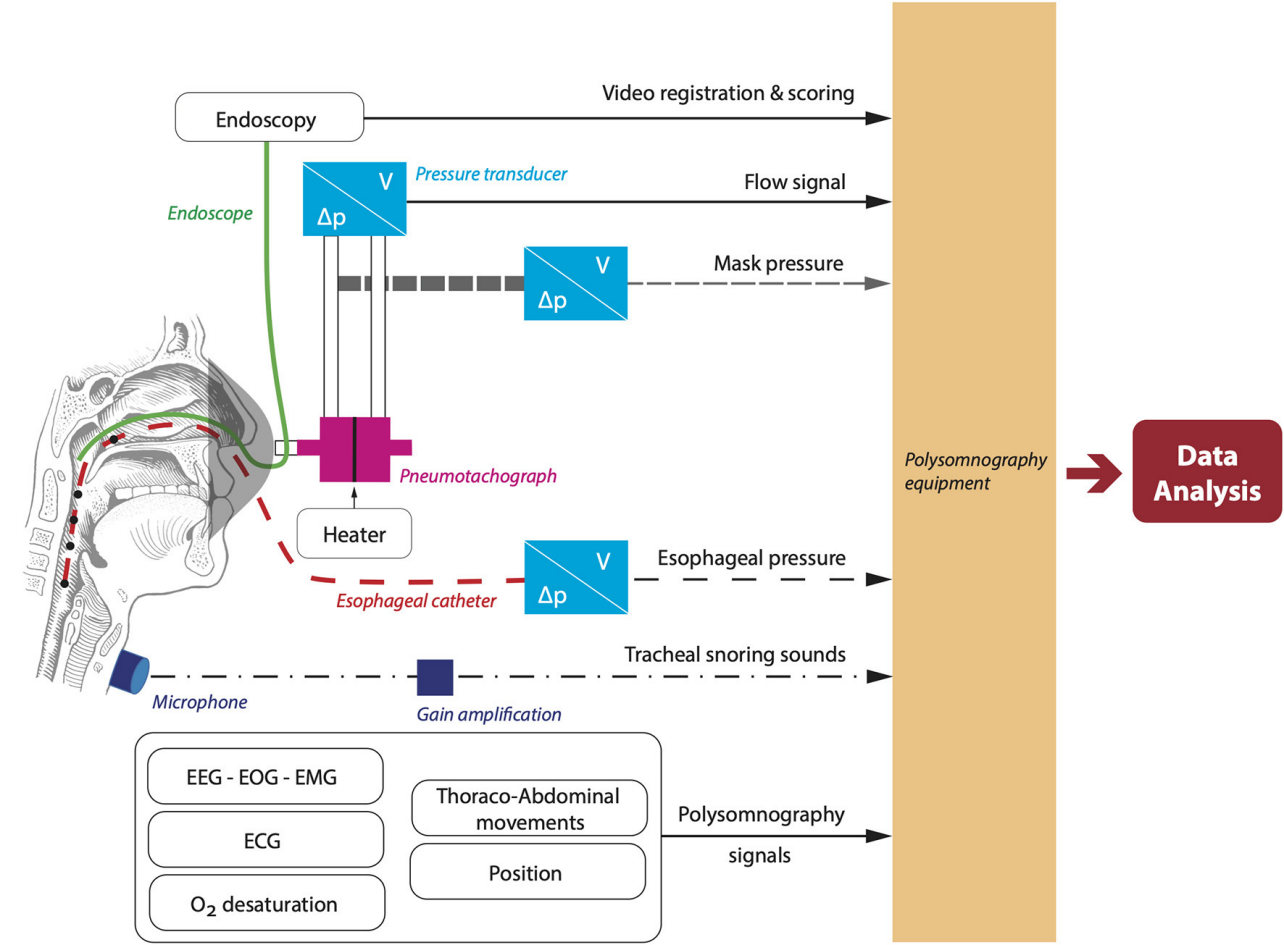
Overview of the set-up to simultaneously measure airflow and capture endoscopy footage. ECG, electrocardiography; EEG, electroencephalography; EMG, electromyography; EOG, electrooculography.

Airway visualization during natural sleep will be achieved by a 2.8 mm diameter pediatric bronchoscope (BF-XP-190, Olympus Europe, Hamburg, Germany) with attached video-processor (CV-190, Olympus Europe, Hamburg, Germany) inserted through an oronasal mask. Before insertion, topical application of a decongestant and topical anesthetic will be applied. The patient will be asked to sleep in the supine position during the measurements. Before sleep onset, the tip of the endoscope will be positioned just above the soft palate. Subsequently, collapse at different upper airway levels will be observed during the night by repositioning the endoscope repeatedly at the naso- and oropharynx. An overview of the set-up is given in Figure 3.

Airflow will be assessed using a calibrated pneumotachograph with heater control (3,700 A pneumotachographs and 3,850 AF pneumotach heater control, Hans-Rudolph, Shawnee, KS, USA) attached to a calibrated pressure and volume transducer with matching carrier demodulator (Validyne Engineering, Northridge, CA, USA). Mask pressure will be measured using a pressure transducer referenced to atmosphere and arterial oxygen will be monitored at the fingertip. Moreover, an esophageal pressure catheter will be inserted into one of the nostrils through a sealed hole in the mask.

During DISE, the airway will be visualized with a flexible nasopharyngoscope (END-GP, 3.7 mm diameter, Olympus Europe, Hamburg, Germany). In addition to the endoscopic video recordings, we will also measure pneumotachograph flow (Hans-Rudolph), calibrated mask pressure, EEG, EOG, chin EMG, SaO_2_, and thoraco-abdominal movements. These additional measurements will be recorded using an integrated Alice 6 LDx system (Philips Respironics, Murrysville, Pennsylvania, USA), synchronized with the DISE video acquisition system (Figure 3).

Additional acoustic measurements will be performed during NSE and DISE to combine tracheal snoring sounds with flow patterns and examine associations with the site(s) of collapse. A calibrated omnidirectional zoom microphone (Sony ECM-77B) will be attached to the trachea. A custom-made holder will keep the microphone in place. Gain will be obtained through an M-track 2 × 2 (M-audio). Sound signals will be integrated into the DISE and NSE set-up to allow for time-coupling of all signals.

### Signal Processing

Several features, including respiratory measures in time and frequency domains, will be extracted from the flow and acoustic signal. The endotypic traits—collapsibility, loop gain, arousal threshold, and muscle responsiveness—will be determined as described previously (49–52). First, ventilatory drive, defined as ventilation during unobstructed breaths, will be calculated as tidal volume × respiratory rate (L.min^−1^) using diaphragm EMG. Determination of the percentage of flow:drive will be done by dividing ventilation (L.min^−1^) by ventilatory drive (L.min^−1^). Collapsibility of the pharynx will be characterized by the ventilation at a normal ventilatory drive (Vpassive) during sleep. Ventilatory control stability is most commonly defined by loop gain, which will be calculated as the ratio of the ventilatory response and the associated ventilatory disturbance (50). The arousal threshold will be measured as the mean ventilatory drive preceding the start of an arousal (51). The increment in ventilation from Vpassive to ventilation at the arousal threshold will be computed, indicating the muscle responsiveness (52).

Individual flow limited breaths will be scored and flow shape analysis will be performed according to Mann et al. (44). A particular focus will be on NED, as this feature was commonly used in previous research to study upper airway mechanics during DISE (27, 30).

Acoustic features from tracheal snores will be estimated primarily in the frequency domains. Tracheal snoring sounds first undergo preprocessing with a high pass filter with a cutoff frequency of 5 Hz. Features are then extracted from the processed snore signal on a window-level with overlap (e.g., 100 ms window with 50% overlap). These features will include those developed for speech analysis that have been adapted for use in analyzing snoring sounds (33, 34, 37, 38). They include, but are not limited to, power in relevant frequency bands (45), formant frequencies, pitch, harmonicity, mel-frequency cepstral coefficients, and several other metrics describing the power spectral density of the snore signal. Time domain features will also be explored, such as zero-crossing rate, energy, snore duration, and within-breath timing of peak snore amplitude. Snores will be evaluated at the breath-level by averaging windows within the bounds of inspiratory start and stop times (for inspiratory snores) and expiratory start and stop times (for expiratory snores) for all breaths. All breath-level features, such as snore features, flow shape features, and ventilation, are stored in a table detailing all breaths for the night (along with event type, sleep state, etc.), which can be used for various analysis applications. These applications include models for predicting the site of collapse from snore sounds and/or flow shapes that can be applied to predict the site of collapse at the breath-level, which can then be utilized to assess likelihood of response to alternative non-CPAP therapies.

The primary site of collapse of each individual flow-limited breath will be labeled using a custom-made scoring tool. Patient level analysis requires summarizing flow shapes and acoustic features for the patient (e.g., mean values of each feature) and using patient level scoring of site of collapse as the outcome. The overall clinical scoring will be used for analysis on a patient level (Figure 2) (25). The comparison of flow shape and tracheal snore features of specific sites of collapse between DISE and NSE (Aim 3), will be performed on both a breath level and a patient level. Further, model validation for predicting site of collapse using flow shape and tracheal snore features (Aim 4) will also be done on both a breath level and a patient level.

### Statistical Analysis

The chi squared test will be performed to compare the site, degree, and pattern of upper airway collapse on a patient level between NSE and DISE (Aim 1). Analysis, linking flow, and acoustic parameters to the OSA traits, will be done on a patient level and on a breath level using Matlab (The MathWorks, Inc., Natick, Massachusetts, USA) (Aim 2). To compare flow shape metrics and acoustic features from snores for each primary site of collapse between DISE and NSE (on a patient level and a breath level), unpaired comparison tests (e.g., *t*-test, Mann-Whitney U-test) will be performed (Aim 3). Lastly, for Aim 4 we will prospectively validate previously developed models (27–31, 44, 45) that predict the site of pharyngeal collapse on DISE and NSE data. Prediction will be compared to the true site of collapse labels generated by expert reviewers, as previously described. Prediction performance between DISE and NSE will be qualitatively compared.

## Discussion

Obstructive sleep apnea is increasingly recognized as a complex and heterogenous disorder in terms of its causes, clinical expression and susceptibility to comorbidities. As a result, a “one-size-fits-all” management approach is not appropriate and should therefore be avoided. A more personalized patient selection through an endotyping approach would allow not only to increase true clinical effectiveness but also to avoid the current “trial-and-error” method which can significantly delay appropriate treatment and lead to a potential waste of resources. Therefore, there is a need to evaluate pathophysiological endotyping in OSA patients, with upper airway collapse at one or more upper airway levels during sleep as one of the main pathophysiological features. Knowing which trait(s) should be addressed in each patient is crucial to predict and achieve response to non-CPAP therapies (53, 54). To assess the site(s) and pattern(s) of upper airway collapse, NSE is defined as the gold standard. Flow shape and tracheal snoring analysis are evolving as non-invasive measurement techniques. Although DISE is used as the clinical alternative to assess the site(s) and pattern(s) of upper airway collapse, associations between DISE and NSE collapse, and associated flow and snoring patterns, have not been quantified satisfactorily.

### Study Protocol

The proposed study protocol has different crucial aspects. A recent clinical PSG no more than 2 years old is needed to confirm OSA diagnosis. Hereby, we prevent patients being included in the study protocol while they do not suffer from moderate to severe OSA. The completion of the research PSG marks the official start for the study patient. During this PSG, baseline gold standard measurements will be performed and coupled with NSE video recordings. After the research PSG, a DISE with flow measurements and tracheal snoring sounds analysis will be scheduled. During both NSE and DISE, flow measurements and acoustic analysis of tracheal snoring sounds will be performed as the latter are assumed to be innovative methods to predict upper airway collapse, in accordance with previous research (33, 34, 42, 43). This comparison not only allows us to compare collapse patterns during NSE and DISE, but also to validate previously developed models for predicting the sites of collapse from airflow and snoring sounds and to compare prediction performance between NSE and DISE.

### Study Aims

Our first aim focuses on the comparison in collapse patters between NSE and DISE. This project will be one of the first studies to formally compare both examinations in each subject. No restriction will be put on the desired treatment for these patients. This is crucial as we aim to collect data in OSA patients with different collapse sites. To allow proper prediction, sufficient data is needed for each site of obstruction. For example, patients with CCCp, as assessed during DISE, are currently excluded for upper airway stimulation therapy (19). Thus, we would exclude this collapse type by only including patients eligible for upper airway stimulation therapy.

Moreover, this will be, to the authors' best knowledge, the first comparative research in flow shape analysis and tracheal snoring sounds analysis between DISE and NSE. As shown by Vanderveken et al. (55), DISE requires an additional step in the clinical pathway. A future goal would be to omit this step in certain patients to achieve a faster screening process resulting in more time- and cost-effective treatment. Upfront response prediction using the combination of flow characteristics and tracheal snoring sounds has the potential to optimize and shorten the clinical pathway. By reducing the clinical pathway and as such reducing costs, OSA treatment will be accessible for a larger patient population, reducing the portion of patients remaining undertreated.

### Limitations

The performance of NSE requires the presence of one or two investigators during a whole night to examine only one to two patients. Moreover, blinding will be attained by having different ENT surgeons performing the DISE and NSE of the same participating patient, which will necessitate another additional researcher. Furthermore, NSE is an invasive study for the patient, accompanied with numerous challenges for the clinicians, such as the risk of awakening the patient when moving the scope to a different upper airway level. However, in previous studies subjects seemed to tolerate scope insertion and were able to sleep during the examination with minimal sleep disruption during manipulation of the endoscope (16, 27–29, 31, 56, 57). Nonetheless, it is a highly time consuming and labor-intensive study, limiting the feasibility to examine many patients in a short time frame and making it a certain challenge to achieve a large sample size.

Natural sleep endoscopy will only be performed in the supine position, which makes it hard to evaluate upper airway collapse in different sleeping positions. However, upper airway collapse is often more pronounced in the supine position, depending on the responsible structure, which makes it a reliable body position for this study (56, 58). During DISE, the upper airway will additionally be evaluated after lateral head rotation. In this way, changes in upper airway collapse related to body position can still be assessed in each patient.

The effect of sedative drugs used during DISE (propofol and midazolam) on upper airway collapse is not fully known. Previous studies showed that respiratory parameters such as the AHI and the mean oxygen saturation remained unchanged during propofol sedation (59). Moreover, the use of propofol did not induce snoring in healthy subjects in earlier publications (59). However, according to previous studies, propofol might lead to a 40% greater decrease in genioglossal muscle activity compared to natural nonrapid eye movement sleep (60, 61) and increasing propofol concentrations were shown to be associated with an increase in collapsibility (62–65). Therefore, the comparison with an endoscopic evaluation during natural sleep might become even more interesting.

Normal sleep at home or during a clinical PSG differs from a research measurement during NSE because of the presence of a pediatric bronchoscope, a sealed mask, an esophageal pressure catheter and a pneumotachograph, which may influence the results. However, according to Maddison et al., a catheter into the pharynx might not have an influence on upper airway collapsibility (66). Therefore, we argue that NSE remains a relatively reliable examination that closely resembles natural sleep under normal conditions.

### Study Set-Up Advantages

By including measurements during both natural and drug-induced sleep, we will be able to compare the results obtained during both conditions. This is of great importance as only limited formal comparison of collapse patterns during natural and drug-induced sleep has been done. Moreover, since CCCp, as assessed during DISE, is currently considered as a formal exclusion parameter for upper airway stimulation therapy, this comparison is of utmost importance to understand OSA pathophysiology.

Second, an esophageal pressure catheter will be added to the set-up, which may improve the accuracy of detecting flow limitation. Therefore, esophageal pressure measurements may play an important role in distinguishing obstructive from central apneas. However, the presence of endoscopy during drug-induced or natural sleep also provides certitude in this differentiation. If the quality of the endoscopic images during natural sleep would be disturbed at certain hours of the night, the esophageal pressure measurements might provide us a back-up in evaluating the presence or absence of upper airway obstruction.

Third, this is an investigator-blinded comparative cohort study with different ENT surgeons conducting DISE and NSE in each patient, thereby minimizing the risk of biased observations.

Fourth, both the NSE set-up and DISE set-up with additional measurements have been successful in preliminary testing and are operational. One of the primary advantages of this dataset is that it enables within-patient comparison of the differences in the site of collapse when under natural and drug-induced sleep. Further, it allows for comparison between DISE and NSE of non-invasive measures of the site of collapse derived from flow shapes and snoring sounds. This would then facilitate the validation of some earlier developed models, based on drug-induced sleep and imply that they are applicable to normal sleep. Furthermore, the finding that gold standard measures (e.g., site of pharyngeal collapse) from DISE and NSE are sufficiently similar, would imply that new non-invasive measures (e.g., flow shape/snoring sound derived site of collapse) could be effectively developed and validated from DISE studies. The key advantage being that DISE studies can be collected more rapidly than NSE studies, will make it feasible to collect large patient datasets required to develop more complex prediction models.

## Conclusion

This project will be one of the first studies to formally compare collapse patterns during natural and drug-induced sleep. Moreover, this will be, to the authors' best knowledge, the first comparative research in airflow shape and tracheal snoring sounds analysis between DISE and NSE. These novel and non-invasive diagnostic methods studying upper airway mechanics during sleep will be simultaneously validated against DISE and NSE.

## Ethics Statement

The studies involving human participants were reviewed and approved by the Institutional Ethics Committee of the University of Antwerp and Antwerp University Hospital (Protocol no. 21/02/009). The patients/participants provided their written informed consent to participate in this study. Written informed consent was obtained from the individual(s) for the publication of any potentially identifiable images or data included in this article.

## Author Contributions

OV, AW, DV, and SO worked on the conception. OV, AW, DV, SO, KV, EV, EK, MW, and JV designed the methodology. KV, EV, DV, and SO contributed to the manuscript first draft. KV drafted the final version of the article. All authors contributed to revision and final approval of the manuscript.

## Funding

This study will be supported by grants from the National Institutes of Health (HL102321 and HL128658) to AW, a Senior Clinical Fellowship Grant (Fundamenteel Klinisch Mandaat) from the Research Foundation Flanders-Vlaanderen (1833517N) to OV, and a postdoctoral fellowship at the Research Foundation Flanders (FWO) (1299822N) to SO.

## Conflict of Interest

AW reports grants and personal fees from SomniFix; grants from Sanofi and fees from Nox, Apnimed, Bayer, and Inspire Medical Systems outside the submitted work. JV reports grants and fees from SomnoMed, AirLiquide, Vivisol, Mediq Tefa, Medidis, OSG, Agfa-Gevaert, Accuramed, Bioprojet, Jazz Pharmaceutics, Desitin, Idorsia, Nightbalance, Inspire Medical Systems, Heinen and Löwenstein, Ectosense, Philips, and ResMed outside the submitted work. OV reports grants from Inspire Medical Systems, Nightbalance, GlaxoSmithKline, and LivaNova at the Antwerp University Hospital outside the submitted work. The funders were not involved in the study design, collection, the writing of this article or the decision to submit it for publication. The remaining authors declare that the research was conducted in the absence of any commercial or financial relationships that could be construed as a potential conflict of interest.

## Publisher's Note

All claims expressed in this article are solely those of the authors and do not necessarily represent those of their affiliated organizations, or those of the publisher, the editors and the reviewers. Any product that may be evaluated in this article, or claim that may be made by its manufacturer, is not guaranteed or endorsed by the publisher.

## Abbreviations

AASM: American Academy of Sleep Medicine
AHI: apnea-hypopnea index
BMI: body mass index
CCCp: complete concentric collapse at the level of the palate
CPAP: continuous positive airway pressure
DISE: drug-induced sleep endoscopy
EEG: electroencephalography
ECG: electrocardiography
EMG: electromyography
EOG: electro-oculography
ENT: ear, nose, throat
HMS: Harvard Medical School
NED: negative effort dependence
NSE: natural sleep endoscopy
OSA: obstructive sleep apnea
PSG: polysomnography.
